# Examining the factors affecting sustained use in smart interactive cabinet design: A comparative analysis using SEM and fsQCA

**DOI:** 10.1016/j.heliyon.2024.e39715

**Published:** 2024-10-22

**Authors:** Chengmin Zhou, Xuechen Zhang, Jake Kaner

**Affiliations:** a College of Furnishings and Industrial Design, Nanjing Forestry University, Nanjing, Jiangsu, China; b Jiangsu Co-innovation Center of Efficient Processing and Utilization of Forest Resources, Nanjing Forestry University, Nanjing, Jiangsu, China; c School of Art and Design, Nottingham Trent University, Nottingham, United Kingdom

**Keywords:** Intelligent cabinet design, Perceived value, fsQCA, Experiential marketing, Usage intention, Structural equation modeling

## Abstract

Understanding the factors that contribute to sustained user use is critical to the design and marketing of smart interactive cabinets, which have gained great popularity in recent years. This study investigates the factors that impact users' intentions to continue using smart interactive cabinets and examines how these factors influence product design and marketing strategies. However, existing research has mainly focused on the development and performance evaluation of smart interaction technologies, with less attention paid to users' continued willingness to use smart interaction cabinets and their determinants. To address this gap, 304 valid samples were randomly collected for empirical analysis and hypothesis testing, and a questionnaire survey was administered to 304 young Chinese urban Chinese users born in the 1980s and 1990s. By synthesizing and analyzing the usage patterns and influencing factors among young consumers, the study employs a combination of covariance-based structural equation modeling (CB-SEM) and fuzzy-set qualitative comparative analysis (fsQCA) to evaluate the data.

The analysis highlights the significance of perceived usefulness, ease of use, and innovation in predicting users' intentions to continue using smart interactive cabinets. This study offers insights into the factors influencing continued use and provides valuable guidance for manufacturers and marketers in designing and marketing smart interactive products to enhance user experience and satisfaction.

## 1 Introduction

The development of information technology in the era of user-centered design has brought about numerous advancements in traditional kitchens and interior furniture [1,2]. The prolonged impact of COVID-19 has resulted in individuals in China becoming increasingly accustomed to spending more time at home. This has led to a growing emphasis on the quality of interior spaces within homes and an increasing demand for a higher quality of life [3]. Consequently, the design focus for future smart kitchens has shifted towards enhancing human-computer interaction, improving user experience, and facilitating the growth of new kitchen technologies. Addressing the psychological expectations of contemporary consumers through effective kitchen design has emerged as a pressing issue. The kitchen is known as the "heart of the family" and has become an important area of family life due to its high frequency of use, intensive facilities, and both multiple functionality and socialization [4]. The continuous improvement of economic consumption level and intelligent technology has led to changes in the characteristics of consumer groups, shifting away from the low price war as the main driver of sales [5]. This evolution has prompted consumers to demand higher quality and advanced technology in kitchen products, driving the renewal of traditional kitchen items [6].

The design of modern kitchens addressing the needs of contemporary users requires a holistic approach considering spatial layout, ergonomics, mental health, and technological orientation to enhance user experiences. The spatial layout and arrangement of functional modules in the kitchen play a vital role in ensuring efficient kitchen work and avoiding issues such as muscle aches [7]. Moreover, accommodating the diverse needs of individuals of different ages when designing a kitchen is crucial [8,9]. To optimize user efficiency, machine learning models can be employed to generate layout recommendations, facilitating scientific and efficient kitchen operations [10]. Recent advancements in kitchen design extend beyond traditional cooking spaces and focus on enhancing family unity and well-being through innovative functionalities. For instance, Ficocelli and Nejat designed an assisted kitchen system utilizing two-way voice communication and automated cabinets to aid older users with cognition challenges [11]. Researchers, such as Sun and Wu [12], have investigated smart kitchens that integrate motion analytics and tangible user interfaces to enhance collaborative cooking experiences, fostering intimacy, communication, education, entertainment, and creativity. The ongoing advancement of technology continues to reshape how we engage with our kitchen environments. While some studies have used WiFi signals to analyze daily human activities and distinguish user needs in smart home interactions [[13], [14], [15]], gestural interaction is the most common form of interaction [16,17]. Almost all requests for information or services for smart appliances are made through finger gestures. This form of interaction is particularly effective in the kitchen as it improves hygiene, efficiency and intuitiveness [18].

It is evident that future kitchens will need to positively influence users' lives by leveraging technology and innovation to improve health and well-being standards [6]. Kitchens nowadays have tried to utilize visual projections and sound effects on spatial surfaces to create kitchen living and cooking atmosphere through different atmospheric themes [19]. The hard surfaces on cabinets are very conducive to supporting tangible interactions for synchronized operations and natural interactions, and they are excellent vehicles for meeting the interactive needs of the kitchen [20]. In recent years, the application of the Internet of Things (IoT) and machine learning in kitchens has improved the convenience of daily life and offers broad prospects for the future development of smart homes. These technologies have enhanced the intelligent features of kitchen appliances, further improving the efficiency of food quality monitoring and energy consumption management. Studies show that smart refrigerators predict food quality, reduce waste and enable remote management through machine learning techniques [21,22]. Semantic parsing technology can convert cooking recipes into machine commands, facilitating the automation of kitchen equipment [23]. However, the current literature on cabinet design still focuses on ergonomics, spatial layout design, and environmentally friendly materials [24,25], and there is limited research on how to design smart interactive cabinets that effectively address the interaction needs within kitchen environments.

At the heart of modern kitchen design, intelligent interactive cabinets have become a central feature. These cabinets not only serve the conventional storage function but also incorporate advanced technologies such as intelligent interconnections and user interfaces, significantly enhancing the overall kitchen experience for users. While traditional cabinets form the foundation of intelligent kitchen design, they often fall short in meeting users' personalized needs [26,27]. As a crucial category of smart home products, intelligent interactive cabinets swiftly exhibit their intelligent capabilities [28].

Intelligent interactive cabinets differ from traditional cabinets in that users no longer select them solely for enhanced cooking and storage experiences. Instead, the interactive features of these cabinets significantly influence users' behaviors and emotions [29]. With the emergence of the COVID-19 pandemic altering family dynamics, smart interactive cabinets have acquired a new role, acting as mediators of enhanced family communication and interaction [30]. Furthermore, the development potential of the smart interactive cabinets industry in China remains substantial, underscoring the need to explore factors that drive continued user adoption. Understanding these factors is essential for grasping user acceptance [31], shaping product design and marketing strategies [32,33], elucidating determinants of purchase intention [34], fostering industry innovation, and promoting sustainability [35]. By improving the functionalities and user experience of smart interactive cabinets, a more profound and lasting engagement with smart home devices is facilitated. This, in turn, technological advancement of the smart home industry, thereby enriching users' lives with more refined and personalized experiences.

However, current research on smart homes in China has overlooked in-depth studies on users' continued usage intentions and influencing factors. The success of the smart interactive cabinet market relies not only on technological innovations and functionalities [36], but also significantly on users' continued usage and purchase intentions [34,37]. Understanding the factors that influence users' ongoing intentions is essential for designing products that align with their needs. There is currently a research gap, particularly in the lack of systematic and empirical studies on smart cabinet users' motivation for continued use, satisfaction, dependence, and integration into daily life [38,39]. Furthermore, while research on spatial layout and material selection has matured, there remains insufficient attention on how consumers' psychological factors influence their long-term use of smart interactive products and their purchasing decisions [40].

Examining the factors that influence users' continued use of smart interactive cabinet designs is informative for manufacturers, designers, market experts, and consumers. Manufacturers are able to adapt their products to better meet user needs based on these insights, increasing user satisfaction and brand loyalty [41]. Such research on intentions for sustainable use drives innovation in technology and design, reveals possible product shortcomings, and leads to improvements in functionality and user interface [42]. Understanding the dynamics of sustained use also enhances the marketability of products, making them more attractive to maintain users' long-term interest. Economic benefits are also an important consideration, as sustained product use reduces the frequency of replacements and upgrades, reduces costs, and improves the economic return to the organization [43]. It helps to support the Sustainable Development Goals (SDGs) by reducing resource waste and environmental impacts by extending the product lifespan [44]. Therefore, investigating the factors affecting sustained use in smart interactive cabinets can optimize product functionality and design, deepen understanding of consumer behavior, and support the development of effective marketing strategies [45]，leading to long-term economic and environmental benefits.

This study seeks to address existing research gaps by first developing a theoretical framework to identify key factors that influence the continued use of smart interactive cabinets. Data were then gathered from 304 consumers through a questionnaire to assess the relationship between these factors and users' willingness to sustain product usage. To account for the complex causal relationships arising from multiple interdependent conditions, a mixed-methods approach was employed. This involved the application of Structural Equation Modeling (SEM) and fuzzy set Qualitative Comparative Analysis (fsQCA) to investigate users' intentions for continued use [46].

## 2 Theoretical background and hypothesis development

Consumers' perceived value and expectation confirmation of smart interactive cabinets are key drivers of continued use, and products that meet or exceed expectations can significantly enhance user satisfaction and loyalty [47]. Habit formation also plays a central role in user acceptance of smart cabinets, and the convenience and efficiency of daily use can significantly increase the attractiveness of the product [48]. In addition, social influence and group norms will have an impact on consumers' purchasing decisions. As a symbol of advanced technology, the social acceptance of smart interactive cabinets directly affects their market acceptance [49]. Personalization options can deepen users' psychological ownership of the product and increase their sense of personal connection and identity, thus increasing the likelihood of continued use [50]. Consumers' perceived risks associated with smart devices, particularly concerns regarding data privacy and security, significantly influence their willingness to continue using these products [51]. Transparent privacy policies and reliable security measures are necessary to alleviate user concerns and enhance trust. Comprehensive consideration of these psychological and social factors is essential for designing smart interactive cabinets that meet market and user needs, and can facilitate the effective interconnection of product design and marketing strategies, ensuring the success and durability of smart interactive cabinets in a competitive market [52].

This research presents a theoretical model grounded in the Theory of Consumer Value (TCV) to examine the various factors that affect the willingness to sustain the use of smart interactive cabinets. These influencing factors are categorized as perceived innovation value (PIV), perceived usefulness value (PUV), perceived ease of use value (PEUV), perceived hedonic value (PHV), and perceived product value (PPV). The aim is to understand how these factors collectively impact consumers' continued use of smart interactive cabinets in their day-to-day lives. This understanding will support innovation in product design, enhance user experience and satisfaction, and ultimately foster the sustainable growth of the smart home market. The aim of this theoretical model is to provide a comprehensive understanding of the psychological and behavioral mechanisms that drive consumers' acceptance and use of smart interactive cabinets. Furthermore, this research intends to establish a scientific basis for developing smart home products and formulating effective marketing strategies.

### 2.1 Theory of consumer value (TCV)

The Theory of Consumer Value (TCV) is an important framework for studying consumer decision-making and behavior. Consumer value theory explains how consumers-evaluate and choose products or services by examining five values: functional, social, emotional, conditional and cognitive [53]. The considerable advancements have been made in the study of consumer value theory, providing greater insight into how various values affect consumer behavior.

Functional and emotional values are considered to be the most influential factors on consumer behavior, especially in terms of brand promise and consumer satisfaction [54,55]. Similarly, social value has been shown to significantly influence consumers' purchase intentions in green product purchasing behavior. Additionally, consumers' ethical identity moderates the relationship between emotional and their green purchasing behavior [56]. In addition, a study of purchase intentions for local food products revealed that emotional, functional, and cognitive values were significantly correlated, while social and conditional values were not significant [57]，the innovativeness and compatibility of e-learning service platforms have been identified as significant predictors of users' behavioral intentions [58]. Environmental self-identity is an important mediator between consumption values and behavioural intentions, suggesting that interventions aimed at enhancing environmental self-identity can be effective in promoting willingness to sustain use [59]. Research on food consumption habits has found the importance of affective value in healthy, unhealthy, and mixed food consumption, and physical activity habits can mediate the relationship between value perceptions and food consumption, highlighting their role in linking perceived values with dietary choices [60].

The wide application and expansion of the theory of consumer value in different fields has validated its effectiveness and importance in explaining consumer behavior. TCV not only provides a theoretical framework, but also offers practical guidance on how firms can use consumer value to influence consumer behavior in their marketing strategies [61]. The theory of consumer value focuses on answering the question, "What factors drive consumers to buy or refrain from purchasing (or using) a specific product? Why do they favor one type or brand over other available options?" To explore these questions, we propose five types of consumer-perceived value within the framework of the Theory of Consumer Value (TCV): perceived innovation value (PIV), perceived useful value (PUV), perceived ease of use value (PEUV), perceived hedonic value (PHV), and perceived product value (PPV).

### 2.2 Research hypotheses

**Fig. 1:**
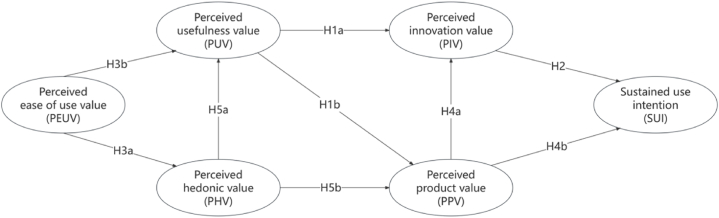
Research model (based on model assumptions).

Perceived usefulness value, defined as the extent to which consumers believe that using a product or service improves their work performance or quality of life, is regarded as a crucial factor influencing their behavioral intentions. Research has shown that consumers' innovative behavior may be enhanced when they perceive a product or service to be highly useful. This perception helps to increase consumer acceptance and willingness to use the new product.This is because perceived value mediates the relationship between consumer innovativeness and the intention to purchase new products [62], and subsequent studies have further confirmed that innovative consumers, when confronted with products that have significant utility, are more willing to pay for and adopt these new products [63]. Today's smart home products not only enhance users' quality of life, but also reflect their social status and image to some extent. Research has shown that there is a potential positive relationship of perceived product innovativeness and perceived social value among consumers. Perceived social value can further contribute to the link between product innovativeness and consumer purchase intention. In addition, Gao, Wang, and Liu (2021) indicated that perceived social value has a significant positive effect on consumers' behavioral intentions (e.g., recommendation intention and revisit intention) [64]. The model tested in this study is presented in Fig. 1. Therefore, we propose the following hypotheses.

Hypothesis 1a (H1a) Perceived usefulness value has a positive effect on perceived innovation value.

Hypothesis 1b (H1b) Perceived usefulness value has a positive effect on perceived product value.

Perceived innovative value describes consumers' willingness to try new products and technologies, and this willingness is recognized as a significant factor influencing the acceptance of these innovations. Research has shown that personality innovativeness indirectly affects continued use intentions through perceived hedonic and utilitarian value. This indicates that consumers with an innovative mindset are more likely to continue using products when they recognize the enjoyment and utility derived from their use [65]. Furthermore, functional innovativeness has a positive impact on behavioral intentions. Specifically, functional innovativeness makes consumers more inclined to accept and continue to use this new delivery service, this further reinforces the positive impact of consumer innovativeness on the intention to continue using the product [66]. Additionally innovative consumers who are able to feel social recognition and pleasure during use are more likely to try and consistently use these new products [67]. Perceived innovation value enhances their willingness to continue using new technologies and products by increasing their perceptions of the pleasure, usefulness and social value of these new technologies and products. Therefore, we propose the following hypotheses.

Hypothesis 2 (H2) Perceived innovation value has a positive impact on the intention to sustain use.

Perceived ease-of-use value refers to how easy users find it to use a technology or product. Users are more likely to experience pleasure in using a technology that feels easy to operate. Perceived ease of use has been shown to significantly enhance the user's hedonic experience, which in turn increases user satisfaction [68]. Perceived ease-of-use value has a significant positive impact on the entertainment perceptions and attitudes of child users, which further demonstrates how ease-of-use can enhance the enjoyment of the usage experience [69]. Users who find a system or application easy to use are more likely to perceive the technology as practically helpful and valuable [70]. This is supported by research by Machdar (2019), which found that information quality significantly influences perceived usefulness through perceived ease of use [71]. This emphasizes how ease of use serves as a mediating variable that influences users' overall evaluation of the technology.

Hypothesis 3a (H3a) Perceived ease of use value has a positive effect on perceived hedonic value.

Hypothesis 3b (H3b) Perceived ease of use value has a positive effect on perceived usefulness value.

Perceived product value is the concept by which consumers comprehensively evaluate the functional, emotional and social dimensions of the benefits provided by a product or service. Perceived value can greatly influence consumer innovative behaviour and the persistence of product use. When consumers perceive a product as having high emotional value and meeting their performance expectations, they are more likely to continue to use the product and refer others to it. This behavior reflects the central role of product value in shaping user satisfaction and loyalty. The emotional value and performance expectations provided by a product are not only key drivers of usage intentions, but also directly contribute to consumers' innovative behavior. This suggests that high perceived value motivates consumers to explore and adopt new products [72]. Meanwhile, a study by Lee et al. (2021) pointed out that perceived hedonic motivation and product compatibility are important predictors of user satisfaction, which in turn influence product usage persistence and recommendation intentions [73]. Furthermore, this is supported by the study of Zhang et al. (2021) who found that perceived high product value enhanced consumers' intention to continue using the product [74]. Perceived value and usefulness significantly influence user satisfaction, which in turn significantly influences intention to continue using. This further illustrates the positive effect of perceived product value on intention to continue using [75]. Therefore, we propose the following hypotheses.

Hypothesis 4a (H4a) Perceived product value has a positive effect on perceived innovation value.

Hypothesis 4b (H4b) Perceived product value has a positive effect on generating willingness to continue using the product.

Perceived hedonic value refers to the pleasure that users experience when interacting with a product or service. Hedonicity also partially moderates the link between the triad of perceived usefulness, perceived ease of use and social value. This suggests that users are more likely to recognize the usefulness role of a product when they find it enjoyable to use [76]. Damanik et al. (2022) state that perceived entertainment significantly influences a user's willingness to continue to use a product by increasing satisfaction, which emphasizes how the entertainment experience can increase a user's perceived usefulness of a produc [77]. Feng and Wei's (2023) also showed that perceived entertainment influences purchase orientation by increasing perceived value, which further demonstrates usefulness of the entertainment experience in increasing the perceived value of a product [78]. Perceived entertainment and perceived usefulness are important antecedents for users to continue to use a product, and perceived entertainment significantly enhances users' value perceptions of a product [79]. We make the following assumptions.

Hypothesis 5a (H5a) Perceived hedonic value has a positive effect on perceived useful value.

Hypothesis 5b (H5b) Perceived hedonic value has a positive effect on perceived product value.

## 3 Methodology

### 3.1 Participants

Recently, renovating smart home software and hardware has become a popular choice for house renovation and transformation [80]. The smart home platform system has been rapidly developed and improved with the advancement of IoT technology. While buying a house provides stability and security, developing a sense of belonging requires emotional investment. As cooking evolves from a chore to a source of enjoyment and emotional interaction, the dining and kitchen space fulfills the emotional and social needs of young consumers [6]. Due to limited housing space, open kitchens have gained popularity among the post-90s generation. They not only expand the living room area but also facilitate interactions with friends and family, making cooking a more enjoyable experience. Nonetheless, the main criticism of smart products on the market stems from their perceived ‘lack of intelligence’ and ‘limited practicality’.

The growth of income levels among Chinese residents has resulted in a significant increase in consumer spending and the demand for personalized home decoration. This trend is further fueled by the emergence of the new generation of consumers born in the 1980s and 1990s, who have a strong preference for high-quality, personalized products and immersive experiences. The acceleration of urbanization also contributes to the increase in consumer spending and demand for personalized home decoration. Given the durable nature of kitchen cabinet products that are not easily replaced, sales of custom kitchen cabinets are often tied to housing sales. The primary homebuying demographic has shifted towards the post-90s generation. Therefore, this study primarily targets individuals born in the 1980s and 1990s, focusing on the user group of intelligent interactive cabinet products. The advancement of smart industries presents companies with opportunities to lower labor costs, enhance productivity, reduce overall expenses, and produce a broader variety of personalized intelligent kitchen cabinet products. It encompasses intelligent design, configuration, and production, aligning with the strong preference for personalized products among the new generation of consumers.

### 3.2 Data collection

In the context of this study, no established scale exists in the current literature, and thus one needed to be developed. To address this, numerous interviews were conducted with cabinet designers and frequent cabinet users prior to finalizing the questionnaire. Several rounds of pre-surveys were also carried out, followed by multiple revisions and refinements to the scale. In May 2023, before collecting a larger set of questionnaires, a small-sample factor analysis was conducted using 60 pre-administered questionnaires. The small sample factor analysis effectively summarized the six components and the results were as expected. The data were analyzed using SPSS 28.0 and AMOS 24.0 and demonstrated well reliability and validity, fulfilling the requirements for practical application.

The official survey was conducted from June to July 2023, and the questionnaire was randomly distributed to the target user group through a Chinese online questionnaire collection platform. The questionnaire, developed by the authors, was divided into three parts, the first of which consisted of introductory and reading materials that allowed participants to understand the survey procedures and the data confidentiality statement. When participants fully understood and agreed to participate in the survey, they could proceed to the subsequent steps. The second section consisted of scale measurement questions, and the third section contained demographic information. The key demographic information (Table 1) showed that the sample was relatively evenly distributed to reflect the overall characteristics of the target consumers.

**Table 1:** Demographic profile.

Demographic variable	Category	N	Percent
Gender	Male	156	51.32 %
	Female	148	48.68 %
Age	24-30	113	37.17 %
	30-35	77	25.33 %
	35-40	92	30.26 %
	40-43	22	7.24 %
Occupation	Students	57	18.75 %
	State-owned enterprises	89	29.28 %
	Private enterprises	137	45.07 %
	Foreign-invested enterprises	21	6.90 %
Education	Junior High School	17	5.59 %
	General High School	46	15.13 %
	College	211	69.41 %
	Undergraduate	15	4.94 %
	Master	9	2.96 %
	PhD	6	1.97 %
Time spent using the cabinet (days of the week)	1–3 days	83	27.30 %
	4–7 days	221	72.70 %

The questionnaire was completed in Chinese. The questionnaire utilized a Likert scale, in which a score of 1 indicates complete disagreement and 7 indicates complete agreement. Table 2 shows the Q1-Q25 of the evaluation questionnaire used for factor analysis. According to the document of the Department of Science and Technology of Nanjing Forestry University1 [2021]. This study received ethical approval from the Ethics Committee of Nanjing Forestry University, with the reference number 20230215004. The data collected is intended solely for academic purposes. Following data collection, 356 questionnaires were retrieved, but invalid responses were excluded, such as those with unusually short response times, duplicates, or incomplete answers. This left 304 valid questionnaires, resulting in a validity rate of 85.39 %. A larger sample size is generally recommended to improve the statistical validity and accuracy of parameter estimates, with a commonly suggested ratio of 20:1, or at least 10:1. Lower ratios may reduce confidence in the results [81]. For our model containing 25 quantitative questions, 304 samples satisfy this requirement, ensuring statistical validity and precision of parameter estimates [82].

**Table 2:** Latent variables, observed variables, along with mean values and standard deviations.

Latent Variable	Observed Variables	M	SD
Perceived innovation value（PIV; Cronbach α = 0.833）			
PIV1	Would you like to use cabinetry with intelligent interaction systems.	5.88	0.798
PIV2	Include a flow design in your cabinet design that can assist in cooking.		
PIV3	Do you want to control your cabinetry in a non-traditional way (e.g., gesture control, voice control).		
PIV4	If the smart cabinet has an interactive display that can help with cooking.		
Perceived usefulness value(PUV; Cronbach α = 0.864)			
PUV1	Smart interactive cabinets can provide relevant guidance information to help users complete the operation.	5.81	0.839
PUV2	The use of smart interactive cabinet screens can meet the need for tutorials, cooking utensils, appliances and other information.		
PUV3	I can be a better cook with a smart interactive cabinet.		
PUV4	Ergonomic smart cabinets can reduce physical fatigue from cooking.		
Perceived ease of use value(PEUV; Cronbach α = 0.853)			
PEUV1	Smart interactive cabinets should be designed for all types of users.	5.57	0.948
PEUV2	Learning to use a smart interactive cabinet does not take much time.		
PEUV3	The location of the smart cabinet interactive display should be within the range of visibility for normal viewing angle.		
PEUV4	The interactive system of the smart cabinet can be used to find the desired items effortlessly.		
Perceived hedonic value(PHV; Cronbach α = 0.834）			
PHV1	Cooking together with family and friends is enjoyable.	5.69	0.919
PHV2	Leisure needs during cooking, such as chatting, listening to English, catching up on TV shows, playing smart interactive cabinets, etc.		
PHV3	When I finish cooking, I need to take and share pictures and videos of the cooking process and results.		
PHV4	I hope the final cooking result can be comparable to the cooking tutorial in terms of visual and taste.		
Perceived product value(PPV; Cronbach α = 0.884）			
PPV1	The interface of the smart interactive cabinet is friendly and beautiful.	5.78	0.841
PPV2	Cabinetry products have interactive features to ensure cooking safety.		
PPV3	Smart interactive cabinets can provide healthy and tasty tutorials while cooking.		
PPV4	The instructions related to the smart interactive cabinet are clear (e.g. set up cooking classes, post relevant news).		
PPV5	Using the Smart Cabinet helps me to share content or exchange experiences with other cooks.		
Sustained use intention(SUI; Cronbach α = 0.865)			
SUI1	In the future, I plan to use the Smart Cabinet to assist with cooking.	5.85	0.871
SUI2	I would be more likely to continue cooking with the Smart Cabinet in the future.		
SUI3	In the future, I will keep using the Smart Cabinet more often, or even increase it.		
SUI4	I would recommend the Smart Interactive Cabinet to friends and family.		
Overall factor Cronbach α	0.915		
KMO	0.901		

In this study, data from 304 questionnaires were processed using SPSS 28.0 software. Factor analysis extracted six factors with a cumulative variance of 63.35 %. The reliability of each factor surpassed the standard threshold of 0.6 [83], with a KMO value of 0.901 (>0.6) and Bartlett's test of sphericity yielding a chi-square of 4088.281, degrees of freedom (df) = 300, and p = 0.000, all meeting the significance requirement (p < 0.05). The standardized factor loading coefficients for the confirmatory factor analysis were all above 0.5 [84], the AVE values were higher than 0.36 [85], and the CR values exceeded 0.6 [86]. Thus, the data demonstrated satisfactory reliability and validity, meeting the criteria for further application.

## 4 Results

### 4.1 SEM reliability and validity tests

We employed the measurement model to assess the factor loadings of various measured components. It is generally recommended that composite reliability should exceed 0.7. The findings of this study confirmed that the composite reliability for all elements met this threshold, indicating the structure's reliability (see Table 3). Moreover, the factor loadings of the indicators within the constructs were statistically significant, with construct reliability above 0.7 and the average variance extracted (AVE) for each construct greater than 0.5. Therefore, the model demonstrated convergent validity, and the results support the convergent validity of each construct.

**Table 3:** Reliability and convergent validity.

Structure			Load of Factor			CR	AVE
	Effect 1	Effect 2	Effect 3	Effect 4	Effect 5		
Perceived innovation value	0.76	0.80	0.73	0.70		0.84	0.56
Perceived usefulness value	0.73	0.80	0.80	0.82		0.87	0.62
Perceived ease of use value	0.81	0.80	0.71	0.77		0.86	0.60
Perceived hedonic value	0.71	0.82	0.78	0.70		0.84	0.57
Perceived product value	0.79	0.73	0.74	0.81	0.82	0.89	0.61
Sustained use intention	0.78	0.75	0.80	0.81		0.87	0.62

### 4.2 SEM fit test

**Fig. 2:**
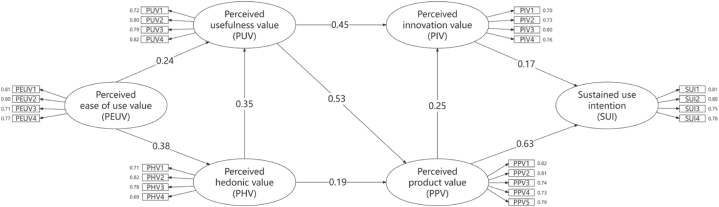
Structural equation modeling results.

In this study, SPSS 28.0 was utilized to analyze the structural equation model for various factors. The overall model fit is summarized in Table 4: The X^2^/df value was 1.502, which is below the standard threshold of 3; RMSEA was 0.04, falling under the acceptable limit of 0.08; and GFI was 0.91, exceeding the minimum requirement of 0.9. These results confirm an acceptable fit. For the incremental fit indices, IFI, TLI, and CFI all scored above 0.9, indicating strong model fit. Additionally, PNFI and PGFI values were 0.80 and 0.75, respectively, both surpassing the minimum of 0.50, further demonstrating good fit. In summary, the data and scale applied in this study show a strong fit with the structural model, and the estimation results demonstrate high reliability. The relationships between factors are illustrated in Fig. 2.

**Table 4:** Fitting index results for the structural equation model.

Inspection Index	Adapt to Standard or Critical Value	Fitted Value	Adaptation Judgment
Absolute fitness index			
X2/df	<3.0	1.50	Yes
RMSEA	<0.05 is excellent; <0.08 is good	0.04	Excellent
GFI	>0.90	0.91	Yes
Value-added adaptability index			
IFI	>0.90	0.97	Yes
TLI	>0.90	0.96	Yes
CFI	>0.90	0.97	Yes
Reduced fitness index			
PNFI	>0.50	0.80	Yes
PGFI	>0.50	0.75	Yes

### 4.3 SEM estimation results

The results of the hypothesis testing are shown in Table 5, where the standardized parameter estimation indicated that all nine hypotheses were statistically significant. The standardized path coefficient between perceived usefulness value and perceived innovation value is 0.45. The coefficient for perceived innovation value's effect on willingness to sustain use is 0.17, while for perceived product value on willingness to sustain use, it is 0.63. Additionally, the standardized path coefficient between perceived ease of use value and perceived hedonic value is 0.38, and the coefficient between perceived product value and perceived innovation value is 0.25. All p-values are below 0.01, indicating that the hypotheses are valid at the 5 % significance level.

**Table 5:** Standardized path coefficients in hypothesis testing by structural equation model.

Hypothesis	Path	Standardized Path Coefficient
H1a	PUV→PIV	0.45∗∗∗
H1b	PUV→PPV	0.53∗∗∗
H2	PIV→SUI	0.17∗∗
H3a	PEUV→PHV	0.38∗∗∗
H3b	PEUV→PUV	0.24∗∗∗
H4a	PPV→PIV	0.25∗∗∗
H4b	PPV→SUI	0.63∗∗∗
H5a	PHV→PUV	0.35∗∗∗
H5b	PHV→PPV	0.19∗∗

∗∗, *p* < 0.01; ∗∗∗, *p* < 0.001.

### 4.4 fsQCA variable selection and calibration

In this study, five variables were selected as antecedent variables. The antecedent conditions were first calibrated for fsQCA analysis. Since the data in the study sample were mainly of the Likert scale numerical type, they needed to be converted to values between 0 and 1 scale accordingly before conducting the analysis. According to the settings of previous similar studies, fsQCA requires values in the range of 0.05–0.95. The process of transforming the Likert scale to 0/1 in the QCA analysis is not strictly homogeneous, but based on the idea of configuration theory in QCA, the closer to 7 the numerical transformation corresponds to the closer to 1 the value in fsQCA is used to reflect the higher probability of its occurrence [87]. Consequently, in this study, the factors influencing the willingness to continue using the product were converted into corresponding probability values based on this criterion, and the transformation was done by transforming the values in the original data with a scale of 1 to 0 and the values with the most scale of 7 to 1, while the values between 1 and 7 were transformed according to the following criteria: 2 → 0.2, 3 → 0.3, 4 → 0.4, 5 → 0.6, and 6 → 0.8. This assignment is based on the reference to the manipulation of data calibration prior to the use of fsQCA method by Y. Liu et al. [88]in the study of users' willingness to adopt government information systems.

### 4.5 fsQCA necessity and sufficiency analysis

Data analysis based on fsQCA begins with an assessment of the necessity of antecedent conditions to understand whether there are antecedent conditions that are consistently present or absent in generating a willingness. Consistency and coverage of the antecedent conditions are key indicators for assessing the necessity of the conditions. Consistency indicates the degree to which a preceding condition (the dependent variable) is essential for producing a specific outcome (the dependent variable). It reflects the probability that the independent variable serves as a necessary condition. Coverage, on the other hand, refers to the percentage of sample cases that encompass the necessary condition variable [89]. When judging necessity, coverage and consistency need to be considered simultaneously, high consistency and high coverage mean that the number of cases presenting the condition is proportional to the number of cases forming the outcome, representing a high level of importance of the necessary condition. High consistency and low coverage indicate that the number of cases that exhibit the condition is not proportional to the number of cases that exhibit the outcome, representing that the necessary condition is not very important. It has been shown that the antecedent variable is judged to be necessary when the threshold of consistency reaches 0.9 or higher.

Table 6 shows that out of the five variables analyzed, the consistency of PIV, PUV and PPV is greater than 0.9, indicating that these three conditions are necessary for the formation of high intention to use, i.e., consumer innovativeness, perceived usefulness and perceived value are necessary to generate high intention to use.

**Table 6:** Analysis of the necessity of high usage intention generation.

Condition tested	Consistency	Coverage
PIV	0.922	0.918
∼PIV	0.275	0.963
PUV	0.910	0.923
∼PUV	0.291	0.958
PEUV	0.858	0.922
∼PEUV	0.346	0.966
PHV	0.882	0.916
∼PHV	0.317	0.969
PPV	0.922	0.940
∼PPV	0.293	0.951

### 4.6 fsQCA conditional combination analysis

Before conducting the histogram analysis, a truth table is created, and a threshold is established to assign a value of 0 to the result variable associated with histograms that show insufficient consistency, indicating that the outcome is absent (see Table 7).

**Table 7:** The truth table.

CI	PU	PEU	ENT	PV	number	UI	raw consist	PRI consist	SYM consist
0	1	0	0	1	2	1	0.999	0.992	0.992
1	1	1	0	1	13	1	0.998	0.995	0.995
1	0	0	1	1	1	1	0.997	0.989	0.989
1	0	0	0	1	1	1	0.997	0.986	0.986
0	1	0	1	1	1	1	0.997	0.984	0.986
1	1	1	0	0	1	1	0.996	0.980	0.980
1	1	0	1	1	23	1	0.996	0.990	0.997
0	0	1	1	1	2	1	0.992	0.961	0.961
0	1	1	1	1	3	1	0.991	0.969	0.976
1	0	1	1	0	1	1	0.991	0.955	0.956
1	0	1	1	1	3	1	0.991	0.968	0.968
1	1	1	1	1	219	1	0.982	0.972	0.994
1	1	1	1	0	6	1	0.982	0.933	0.933
0	0	0	0	0	4	0	0.947	0.637	0.643

To investigate how the combination of paths affects the adequacy of the results, we established a case frequency threshold of 1. The initial minimum threshold for acceptable consistency in path normalization analysis was set at 0.8 [89], while the PRI consistency exceeded 0.75. This resulted in three solutions: the complex solution, the compact solution, and the intermediate solution. In this research, we selected the intermediate solution to explain the outcome variables and constructed an intermediate solution model, as there was reasonable evidence indicating that the results exhibited moderate complexity. The details of the final configuration results are presented in Table 8.

**Table 8:** Configuration assessment for generating sustained willingness to use.

Configurations	1	2	3	4	5
PIV	●		●	●	
PUV		●	●		
PEUV	ⓧ	ⓧ	●	●	●
PHV		●		●	●
PPV	●				●
Consistency	0.994	0.994	0.969	0.963	0.973
Raw coverage	0.343	0.341	0.806	0.787	0.788
Unique Coverage	0.003	0.001	0.033	0.004	0.016
Solution consistency	0.955				
Solution coverage	0.860				

In the table, ● means core antecedent condition is present, ● means edge antecedent condition is present, ⓧ means core antecedent condition is missing, ⊗ is edge antecedent condition is missing, and blank means irrelevant condition.Predecessor configurations sharing the same core condition were grouped into a single category, resulting in the identification of five distinct patterns that influence the willingness for sustainable use. This model demonstrated a strong explanatory effect, with an overall coverage of 0.860 and an overall consistency of 0.955.

Configurations 1 and 2 lack models for perceived ease of use value. The predecessor configuration for pattern one primarily consists of “PIV, ∼PEUV, PPV” In this configuration, both perceived innovation value (PIV) and perceived product value (PPV) are considered core variables, while perceived ease of use value (PEUV) acts as an auxiliary variable. This pattern exhibits a consistency of 0.994, a raw coverage of 0.343, and a unique coverage of 0.003. Here, PIV and PPV collaboratively enhance the formation of high user willingness to utilize the product. Pattern two's predecessor configuration is “PUV, ∼PEUV, PHV” where perceived useful value (PUV) and perceived hedonic value (PHV) are core variables, with PEUV serving as an auxiliary variable. This configuration has a consistency of 0.994, a raw coverage of 0.341, and a unique coverage of 0.001. In this case, perceived useful value and perceived hedonic value have a stronger influence, while perceived ease of use is less impactful. Thus, weakened perceived ease of use affects the willingness to sustainably use smart interactive cabinets.

The core variable in the perceived ease of use value model is PEUV. The predecessor configuration for pattern three is “PIV, PUV, PEUV” which includes PIV, PUV, and PEUV as core variables, resulting in a consistency of 0.969, a raw coverage of 0.806, and a unique coverage of 0.033. When perceived innovation value, perceived useful value, and perceived ease of use value are strong, they consistently generate a willingness to use smart interactive cabinets.Pattern four's predecessor configuration is “PIV, PEUV, PHV” with PIV and PEUV as core variables and PHV as an edge variable. This configuration has a consistency of 0.963, a raw coverage of 0.787, and a unique coverage of 0.004. In this case, perceived innovation value and perceived ease of use value significantly contribute to users' willingness to continue using the product, while perceived hedonic value serves as a marginal condition that plays a secondary role in enhancing this willingness.For pattern five, the predecessor configuration is “PEUV, PHV, PPV” where PEUV and PPV are core variables, and PHV is an edge variable. This configuration shows a consistency of 0.973, a raw coverage of 0.788, and a unique coverage of 0.016. Here, perceived product value and perceived ease of use value are key drivers in establishing users' willingness to continue using the product, with perceived hedonic value playing a supplementary role in enhancing this willingness.

## 5 Discussion

### 5.1 Synergistic insights from SEM and fsQCA

This study seeks to examine the influence of perceived value factors on the willingness to continue using smart interactive cabinets, as well as the interrelationships among these factors, utilizing survey data collected from users of smart interactive cabinets. To achieve this, we will employ both qualitative and quantitative analytical methods to analyze the pathways formed by different combinations of perceived value factors that impact the willingness to sustain usage.

Previous studies have shown that fsQCA findings can effectively complement SEM analyses, providing insights into the complexity of the results [90]. First, perceived usefulness value significantly enhances user acceptance of innovation and product value in smart interactive cabinet design. This suggests that usefulness is a key factor in driving product acceptance and continued use. In smart home product design, users have high expectations for the functionality and practical application value of devices. Smart interactive products with personalized and localized features increase consumers' willingness to sustain use by enhancing perceived product value [91]. In addition, fsQCA results show that when perceived usefulness value and perceived entertainment value are combined, they together significantly increase users' intention to use, emphasizing the importance of utility in product design that combines both utility and pleasure, especially among consumer segments in the target market that seeks an integrated experience [92].

Innovativeness is a key attribute that attracts users to continue interacting and using. Incorporating novel features and technologies in the design of smart interactive cabinets can be effective in increasing users' continued willingness to use. Users' personal innovativeness and perceived hedonic value can enhance acceptance and continued willingness to use smart home devices [93]. Meanwhile, fsQCA results show that when perceived innovation value is combined with perceived product value, this configuration increases willingness to use among consumer groups, demonstrating the utility of innovative elements in increasing product attractiveness and market competitiveness [94]. In addition, perceived ease-of-use value indirectly increases the attractiveness of the product by enhancing the user's operational experience, as well as perceived hedonic value and perceived usefulness value. The importance of ease of use cannot be ignored in smart interactive cabinet design. Users expect to be able to operate and maintain these cabinet products with ease, thereby increasing satisfaction with use and willingness to continue using them. Research in similar smart products has shown that perceived ease of use is directly related to user satisfaction, this characteristic has been identified as a crucial factor in enhancing user satisfaction and encouraging ongoing willingness to use [95].

The SEM analysis revealed that perceived product value has a direct and positive effect on both perceived innovation value and the willingness to continue using the product. This finding indicates that product value is a crucial element in the decision-making process of users, significantly influencing their overall satisfaction and acceptance of the product. When users perceive high product value, it not only enhances their view of the product's innovativeness but also directly supports their intention to continue using it. In the context of designing smart interactive cabinets, a strong perceived product value can foster user loyalty and satisfaction. Additionally, the significance of product value is underscored by its substantial effect on purchase intention, alongside brand perception, in the realm of smart home products [96]. fsQCA results show that the combination of perceived product value and perceived ease-of-use value emerged as the key configuration that drove intention to use. This configuration emphasizes that user satisfaction and loyalty are significantly increased when the product is not only perceived as high value but also easy to use. This is particularly important in modern product design as users increasingly value products for immediate gratification and long-term value [96]. Consumer demand for instant gratification is a direct representation of the need for perceived hedonic value of products in today's society. Perceived hedonic value demonstrated a significant positive effect on perceived usefulness value and perceived product value, suggesting that hedonic value not only directly enhances the user's pleasure with the product, but also enhances the perceived usefulness and overall value of the product. The fun experience that users get when using smart cabinets translates into positive evaluations of product functionality, highlighting the importance of design elements that bring pleasure to users. fsQCA results show that the combination of perceived hedonic value and perceived usefulness value was found to significantly enhance users' willingness to use. It suggests that the attractiveness and likelihood of continued use of a smart interactive product increase significantly when it can satisfy both users' utility and entertainment needs. In addition, although perceived hedonic value usually appears as a marginal condition, the interaction between perceived ease-of-use value and perceived innovative value continues to significantly influence users' willingness to use in particular contexts.

### 5.2 Theoretical implication

This study contributes three significant insights to the field of smart interactive cabinet research and design.

Firstly, our findings reinforce the critical roles of usefulness and ease of use in maintaining user engagement with smart interactive cabinets. Perceived usefulness boosts users' perceived value, indirectly encouraging ongoing use [97]. In simpler terms, both perceived ease of use and perceived usefulness directly influence user satisfaction and their willingness to keep using smart interactive cabinets. Moreover, the perception of usefulness alone is enough to motivate subsequent consumer behavior [98]. Products with excellent ease of use offer intuitive and hassle-free operations, enhancing user satisfaction and streamlining goal achievement while reducing learning barriers and misuse risks. Consequently, ease of use is a significant factor in driving user experience and satisfaction, ultimately fostering continued product usage, thereby enhancing consumer loyalty in competitive markets. Manufacturers can amplify this effect by simplifying user interfaces, providing comprehensive tutorials, and maintaining 24/7 customer support to ensure a smooth and satisfying user experience, which in turn fosters customer loyalty in competitive markets.

Second, consumer innovativeness appeared three times as a core antecedent condition in the fsQCA analysis, which reinforces the fact that future smart interactive products focus as much on the hedonic nature of users experiencing the product, and that experiencers involved in the smart interactive product experience use feelings rather than thoughts in summative judgments, which ultimately creates the conditions for the willingness to continue to use the interactive product. This also suggests that when considering a product from the perspective of the user interaction experience, users may take emotional rather than functional outcomes as a higher goal to pursue. Innovative consumers, particularly those driven by hedonic motivations, are crucial in the adoption of new technologies，including smart interactive cabinets. Their desire for pleasure and social recognition leads them to explore and engage with new product features actively. As a personality trait, innovativeness encompasses various motivational elements behind consumer behavior, such as pleasure-seeking hedonic innovativeness, social, cognitive, and functional motivations. Previous research has shown that attitude significantly influences understanding and intention [99]. Notably, targeting innovators—especially those motivated by hedonic needs—is essential for promoting the adoption of innovative products. Although the results suggest that perceived value leads to user satisfaction and has an independent positive effect on willingness to continue using, as indicated in previous literature [74]. Furthermore, the significance of consumer innovativeness in motivating users to explore new product features and interactive characteristics cannot be understated. Although there is a positive correlation between perceived value and user satisfaction, as well as the intention to continue using a product, consumer innovativeness encourages users to interact with new technologies and proactively seek information to enhance their understanding and knowledge of these innovations [100]. This tendency to explore can lead to more favorable attitudes towards new technologies and, subsequently, to an increased intention to adopt them. By targeting these consumers, companies can not only boost initial product adoption but also sustain long-term engagement by continually stimulating their interest and interaction with innovative features.

Thirdly, the study explored the social dimension of perceived value, perceived entertainment, but found it to be insignificant in influencing sustained use. The reason for our non-significant results may be that users do not seek social approval or recognition from others when using smart interactive cabinets, at least on the smart interactive cabinets we studied here. These traits include expressions like "offers social approval," "enables acceptance by others," "boosts my popularity with family and friends," and "helps me receive affirmation from others through the use of smart interactive products." In the context of media interaction, the frequency of use and hedonic value are regarded as essential factors that strengthen users' intentions to persist in using interactive media [101]. The results of the configurational analysis suggests that unlike digital platforms, smart interactive cabinets may not rely on social approval or recognition to retain users. Instead, the focus should be on creating enriching and enjoyable experiences that meet users' hedonic expectations. To enhance this aspect, integrating social media functionalities to allow for sharing experiences, partnering with content providers for entertainment options, and fostering online and offline user communities could prove effective. These strategies will not only enhance the user's entertainment experience but also support a strong sense of community and belonging, which are pivotal in the sustained use of interactive products.

### 5.3 Marketing implications

Our research offers valuable insights for manufacturers and marketers of smart interactive cabinets in various aspects. First, we propose a straightforward and effective framework to assess the factors influencing users' willingness to engage with smart interactive products. By incorporating clear, measurable attributes, this framework reduces the cognitive load on users and increases their acceptance. Moreover, there is scope for future improvements to this framework. Manufacturers or marketers of cabinets could enhance it by broadening the range of attributes that evaluate perceived and affective value. This framework can also be utilized to analyze the value co-creation processes that occur between producers and consumers, as well as among consumers in the context of smart interactive products.

We have identified socialization and enjoyment as key perceptual dimensions that influence user engagement with these products. Manufacturers can accentuate these dimensions by creating designated zones within the cabinets that encourage interaction across different features. This can be further supported by providing clear, accessible information and ensuring that staff are approachable and well-trained. Moreover, encouraging users to share their experiences on social media can amplify the social and emotional value derived from these products, potentially leading to greater user engagement and product loyalty. In the design of smart interactive cabinets, In the design of smart interactive cabinets, it is crucial to balance practical utility with the user's social and emotional needs. During the initial design stage, it is beneficial to segment the cabinet into various functional zones tailored to distinct activities such as entertainment, work, and social interaction. To amplify the social appeal of the product, incorporating social media functionalities into the cabinet's interface could be considered. This would allow users to effortlessly share content from the cabinet to their social networks, enhancing their engagement and emotional connection through social feedback. By adopting these strategies, manufacturers can boost the marketability of their products and fine-tune features based on real user feedback, ensuring the product aligns seamlessly with consumer lifestyles and preferences.

### 5.4 Limitations of the study and future research

Our research predominantly employs a quantitative approach, assessing the individual and combined effectiveness of Structural Equation Modeling (SEM) and fuzzy-set Qualitative Comparative Analysis (fsQCA) to evaluate perceived value and sustained intention to use, as well as their influence on cabinet satisfaction. While our findings contribute valuable insights, several limitations should be noted. Firstly, the scope of our study is restricted to interactive smart cabinets. Therefore, future research should examine the effectiveness of perceived attributes across different products and contexts. Additionally, alternative research methods, such as qualitative in-depth interviews or focus group discussions, could be employed to gain insights into users' implicit interpretations of perceived attributes and their ongoing intention to use. This direction will enhance the generalizability of perceived attributes in shaping user behavior concerning smart interactive cabinets. Expanding the sample size, validating the model's generalizability, and utilizing diverse research designs could further enrich the understanding of this topic. We recommend conducting additional quantitative studies with product samples from various domains and usage scenarios to validate the proposed research model and identify potential influencing factors. Such efforts will provide deeper insights and guidance for the design and marketing of smart interactive products.

## 6 Conclusion

This study offers a thorough examination of the factors that affect consumers' willingness to continue using smart interactive cabinets through the integration of Structural Equation Modeling (SEM) and fuzzy-set Qualitative Comparative Analysis (fsQCA). It uncovers the intricate relationships among perceived innovation value, perceived usefulness value, perceived ease of use value, perceived hedonic value, and perceived product value. The findings highlight the pivotal role of perceived innovation value in fostering ongoing use, while also acknowledging the dual function of perceived hedonic value. By addressing diverse user needs, this research presents a fresh perspective on the design and marketing of smart home products. It not only contributes new theoretical insights into the factors driving the usage of smart interactive cabinets but also offers practical recommendations for enhancing product appeal and market competitiveness. Additionally, it sets a foundation for future research and product development. This study underscores the significance of integrating these factors into the design and marketing strategies for smart interactive cabinets, providing both theoretical and practical guidance for continuous innovation and optimization of the user experience in smart home products. By deepening our understanding of how these key elements interact to influence users' willingness to continue using cabinets, manufacturers and designers can better align their offerings with market demands, increase market acceptance, and ensure the sustained success of smart cabinetry products.

## Funding

This work was supported by the 2020 Jiangsu Postgraduate International Smart Health Furniture Design and Engineering project (Grant No.202002).This work was also supported by the 2022 International Cooperation Joint Laboratory for Production,Education,Research,and Application of Ecological Health Care on Home Furnishing(Grant No.20220602) and the Qing Lan Project (Grant No.2022QL06).

## CRediT authorship contribution statement

**Chengmin Zhou:** Writing – original draft, Conceptualization. **Xuechen Zhang:** Writing – original draft, Methodology, Formal analysis, Data curation. **Jake Kaner:** Writing – review & editing, Supervision.

## Data availability

Data will be made available on request.

## Declaration of Competing Interest

The authors declare that they have no known competing financial interests or personal relationships that could have appeared to influence the work reported in this paper.
